# Genomics of response to porcine reproductive and respiratory syndrome virus in purebred and crossbred sows: antibody response and performance following natural infection vs. vaccination

**DOI:** 10.1093/jas/skab097

**Published:** 2021-03-29

**Authors:** Leticia P Sanglard, Felipe M W Hickmann, Yijian Huang, Kent A Gray, Daniel C L Linhares, Jack C M Dekkers, Megan C Niederwerder, Rohan L Fernando, Joseph Braccini Neto, Nick V L Serão

**Affiliations:** 1 Department of Animal Science, Iowa State University, Ames, IA 50011, USA; 2 Department of Animal Science, Universidade Federal do Rio Grande do Sul, Porto Alegre, Rio Grande do Sul, 91540-000, Brazil; 3 Smithfield Premium Genetic, Rose Hill, NC 28458, USA; 4 Department of Veterinary Diagnostic and Production Animal Medicine, Iowa State University, Ames, IA 50011, USA; 5 Department of Diagnostic Medicine/Pathobiology, Kansas State University, Manhattan, KS 66506, USA

**Keywords:** genetic correlation, major histocompatibility complex, PRRS, reproductive performance, swine

## Abstract

Antibody response, measured as sample-to-positive (S/P) ratio, to porcine reproductive and respiratory syndrome virus (**PRRSV**) following a PRRSV-outbreak (*S/P*_Outbreak_) in a purebred nucleus and following a PRRSV-vaccination (*S/P*_*Vx*_) in commercial crossbred herds have been proposed as genetic indicator traits for improved reproductive performance in PRRSV-infected purebred and PRRSV-vaccinated crossbred sows, respectively. In this study, we investigated the genetic relationships of *S/P*_Outbreak_ and *S/P*_*Vx*_ with performance at the commercial (vaccinated crossbred sows) and nucleus level (non-infected and PRRSV-infected purebred sows), respectively, and tested the effect of previously identified SNP for these indicator traits. Antibody response was measured on 541 Landrace sows ~54 d after the start of a PRRSV outbreak, and on 906 F1 (Landrace × Large White) gilts ~50 d after vaccination with a commercial PRRSV vaccine. Reproductive performance was recorded for 711 and 428 Landrace sows before and during the PRRSV outbreak, respectively, and for 811 vaccinated F1 animals. The estimate of the genetic correlation (*r*_g_) of *S/P*_Outbreak_ with *S/P*_*Vx*_ was 0.72 ± 0.18. The estimates of *r*_g_ of *S/P*_Outbreak_ with reproductive performance in vaccinated crossbred sows were low to moderate, ranging from 0.05 ± 0.23 to 0.30 ± 0.20. The estimate of *r*_g_ of *S/P*_*Vx*_ with reproductive performance in non-infected purebred sows was moderate and favorable with number born alive (0.50 ± 0.23) but low (0 ± 0.23 to −0.11 ± 0.23) with piglet mortality traits. The estimates of *r*_g_ of *S/P*_*Vx*_ were moderate and negative (−0.38 ± 0.21) with number of mummies in PRRSV-infected purebred sows and low with other traits (−0.30 ± 0.18 to 0.05 ± 0.18). Several significant associations (*P*_0_ > 0.90) of previously reported SNP for S/P ratio (ASGA0032063 and H3GA0020505) were identified for S/P ratio and performance in non-infected purebred and PRRSV-exposed purebred and crossbred sows. Genomic regions harboring the major histocompatibility complex class II region significantly contributed to the genetic correlation of antibody response to PRRSV with most of the traits analyzed. These results indicate that selection for antibody response in purebred sows following a PRRSV outbreak in the nucleus and for antibody response to PRRSV vaccination measured in commercial crossbred sows are expected to increase litter size in purebred and commercial sows.

## Introduction

In swine breeding, selection of genetically superior animals is mostly performed in purebred pigs in the nucleus, with the goal of improving performance of crossbred pigs at the commercial level. However, this selection strategy is less than optimum because the genetic correlation (*r*_g_) between purebred and crossbred performance is less than unity (Wientjes and Calus, 2017). Also, nucleus herds are managed to maximize biosecurity, reducing the exposure of pigs to pathogens and other stressors, limiting the expression of immune-related traits. Therefore, selecting animals for such traits depends on collecting crossbred data at the commercial level and using this information to estimate breeding values for nucleus animals.

Total antibody response to porcine reproductive and respiratory syndrome (**PRRS**) virus (**PRRSV**), measured as sample-to-positive (**S/P**) ratio, has been proposed as an indicator trait for improved reproductive performance in PRRSV-exposed sows (Serão et al., 2014; Sanglard et al., 2020). Following a PRRSV outbreak, Serão et al. (2014) observed that S/P ratio had high heritability (*h*^2^ = 0.45) and high *r*_g_ with reproductive performance in PRRSV-infected sows [0.73 ± 0.23 with number of born alive (**NBA**)]. However, waiting for a PRRSV outbreak to happen for data collection limits the use of this indicator trait in pig breeding schemes. Sanglard et al. (2020) investigated the use of S/P ratio to vaccination in commercial gilts as an effective strategy to continuously generating S/P ratio data in commercial settings. These authors reported a moderate *h*^2^ (0.34 ± 0.05) and high *r*_g_ of S/P ratio to modified live PRRSV vaccine with NBA (0.61 ± 0.16) in the absence of a PRRSV outbreak. These results further support the use of S/P ratio to PRRSV after an outbreak or vaccination as a genetic indicator trait for improved reproductive performance in PRRSV-infected purebred sows and crossbred sows, respectively.

The major histocompatibility complex (**MHC**) region on *Sus scrofa* chromosome (**SSC**) 7 has been shown to control a large part of the genetic variation of S/P ratio. Serão et al. (2014) and Sanglard et al. (2020) reported that this region explained 30% and 15% of the genetic variance of S/P ratio to PRRSV outbreak and vaccination, respectively. This region has also been previously associated with reproductive performance in non-infected pigs (Jung et al., 1989; Vaiman et al., 1998), including PRRSV-vaccinated gilts (Sanglard et al., 2020). Also, Sanglard et al. (2020) showed that this region explained between 25% and 90% of the covariance between S/P ratio and subsequent farrowing performance, further indicating that these traits are, in part, simultaneously controlled by the MHC region. The MHC is a gene-rich region including several immune-related genes. This region can be divided into three, including class I, II, and III genes (Hammer et al., 2020). For example, MHC class II and transporter genes, such as *TAP1* and *TAP2*, have been proposed as candidate genes for S/P ratio in pigs (Serão et al., 2014; Hess et al., 2018; Sanglard et al., 2020).

These results show that S/P ratio to PRRSV is a promising indicator trait for identifying genetically superior animals for improved reproductive performance, regardless of whether sows are vaccinated or naturally infected with PRRSV. However, the potential impact of selecting purebred pigs based on S/P ratio following a PRRSV outbreak on crossbred performance is not known. Likewise, the potential impact of selecting purebred pigs based on S/P ratio to PRRSV vaccination collected in crossbred pigs on purebred performance is unknown. Hence, we proposed to investigate the genetic relationships between S/P ratio and performance in two populations of pigs: a purebred population that underwent PRRSV outbreak and a crossbred population that had been vaccinated to PRRSV.

## Material and Methods

**Figure 1.**
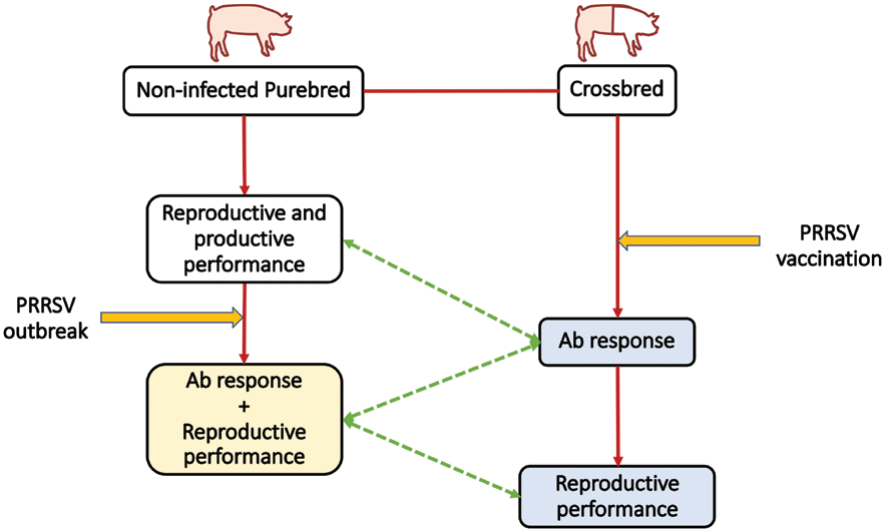
Schematic representation of the animals (purebred and crossbred), data recorded (productive and reproductive performance, and antibody response), and events [PRRSV outbreak and vaccination] included in this study. The white, blue, and yellow boxes represent the non-PRRSV-exposed (i.e., clean), PRRSV-vaccinated, and PRRSV-naturally infected conditions, respectively, when the data were collected. The green dashed line represents the genetic correlations estimated in this study. The red arrows represent the direction of events.

All methods described in this study were approved by the Institutional Animal Care and Use Committee at Iowa State University (**ISU**; IACUC# 6-17-8551-S). Animals from the 2 datasets used in this study belonged to the same breeding company. A schematic representation of the data used in this study is shown in Figure 1.

### Purebred phenotypic data

Typical clinical signs of PRRSV infection, such as decrease of the reproductive performance, were detected in the purebred nucleus during the Spring of 2018. Approximately 54 d after the PRRSV outbreak started, blood samples were collected from 428 Landrace sows (1.5 ± 0.6 years of age) for total antibody response measurement, as sample-to-positive (**S/P**) ratio, by ELISA (IDEXX PRRS X3 Ab Test, Westbrook, Maine) at the Veterinary Diagnostic Laboratory (**VDL**) at ISU (Ames, Iowa). The field PRRSV strain was sequenced and identified as PRRSV 1-7-4, a highly pathogenic strain. The PRRSV outbreak phase was identified based on a combination of methodologies previously described by Lewis et al. (2009), Putz et al. (2019), and Scanlan et al. (2019) as described by Hickmann et al. (2020). The PRRSV outbreak period lasted 16 and 20 wk for mortality and survival litter size traits, respectively. From now on, we will be referring to S/P ratio following the PRRSV outbreak as ***S/P****_**Outbreak**_*.

Four hundred and twenty-eight Landrace sows had records for reproductive performance during the PRRSV outbreak on NBA, number of stillborn (**NSB**), and number born mummified (**MUM**). Number born dead (**NBD**) was calculated as the sum of MUM and NSB, and total number born (**TNB**) was calculated as the sum of NBA and NBD. Of these, 220 sows also had information on body composition collected at 159 ± 5 d of age such as ultrasound measurements of loin muscle depth (**LMD,** cm), intramuscular fat percentage (**IMF,** %), and backfat (**BF,** cm). Ultrasound images were recorded with Aloka 500 ultrasound machine (Corometrics Medical Systems, Wallingford, CT), and IMF was analyzed using the BioSoft Toolbox II Software (Biotronics Inc., Ames, IA). Average daily gain (**ADG**, kg/d) was calculated as the difference between body weight at the end of the finishing period (offtest, 159 ± 5 kg and 159 ± 5 d) and birth weight (1.7 ± 0.3 kg) divided by age at offtest. This dataset will be referred to as ***P****_**Pure_outbreak**_* (performance in PRRSV-infected purebred sows). Reproductive performance data were also available from this herd before the outbreak on 465 Landrace sows (1,115 observations from up to parity 8; 245 sows overlapping with the sows that overwent PRRSV outbreak) from June 2016 to April 2018. Of these, 463 sows also had information on the aforementioned body composition and growth traits. This dataset will be referred to as ***P***_***Pure_clean***_ (performance in non-infected purebred sows). Summary statistics for the purebred/PRRSV outbreak data are presented in Table 1.

**Table 1.** Mean, standard deviation (SD), minimum value (Min), maximum value (Max), and number of individuals (*N*) in the data

Trait^1^	Mean	SD	Min	Max	*N*
*S/P* *_Outbreak_*	1.22	0.31	0.19	2.08	545
*S/P*_*Vx*_	1.41	0.45	0.06	2.55	906
*P* *_Pure_clean_* ^2^					
ADG, kg/d	0.76	0.06	0.58	1.09	463
LMD, cm	4.53	0.58	3.07	6.30	463
IMF, %	2.17	0.72	0.30	4.28	463
BF, cm	1.21	0.36	0.48	3.02	463
NBA	12.03	3.38	0	21	465
NSB	0.85	1.25	0	8	465
MUM	0.44	0.84	0	6	465
NBD	1.30	1.58	0	11	465
TNB	13.32	3.93	2	24	465
*P* *_Pure_outbreak_*					
ADG, kg/d	0.76	0.06	0.54	1.09	220
LMD, cm	4.79	0.67	3.07	6.83	220
IMF, %	2.16	0.76	0.30	7.17	220
BF, cm	1.23	0.34	0.48	3.02	220
NBA	7.50	4.73	0	19	428
NSB	1.26	0.9	0	12	428
MUM	1.53	1.78	0	18	428
NBD	4.17	4.07	0	12	428
TNB	12.87	4.03	3	24	428
*P*_*Cross_Vx*_^3^					
NBA	11.62	3.01	0	22	811
NSB	0.48	0.92	0	10	811
MUM	0.36	1.02	0	13	811
NBD	0.86	1.46	0	13	811
TNB	12.48	2.89	2	24	811

^1^Traits: *S/P_Outbreak_*, antibody response to porcine reproductive and respiratory syndrome (PRRS) virus (PRRSV); *S/P*_*Vx*_, antibody response to PRRSV vaccination; *P_Pure_clean_*, performance of non-infected purebred sows*; P_Pure_clean_*, performance of non-infected purebred sows; *P*_Pure_outbreak_, performance of PRRSV-infected purebred sows; *P*_*Cross_Vx*_, performance of crossbred PRRSV-vaccinated sows; ADG, average daily gain; LMD, loin muscle depth; BF, backfat; and IMF, intramuscular fat percentage; NBA, number born alive; NSB, number stillborn; MUM, number mummies; NBD, number born dead; TNB, total number born.

^2^Each animal had data for up to parity 8 for a total of 1,116 observations.

^3^Each animal had data for up to parity 3 for a total of 1,809 observations.

### Crossbred phenotypic data

A full description of the crossbred vaccinated animals used in this study is by Sanglard et al. (2020). Briefly, 906 F1 (Landrace × Large White) replacement gilts from 2 commercial farms in North Carolina were vaccinated (139 ± 17 d old) intramuscularly with a commercial modified life PRRSV vaccine (Ingelvac PRRS MLV, Boehringer Ingelheim Animal Health, Ames, IA). These animals were predominantly half-sibs of the Landrace purebred population described above. Blood samples were collected at ~50 d (52- and 53-d postvaccination for one farm, and 46 d postvaccination for the other farm) after vaccination in 3 contemporary groups (**CG**; days of blood collection). Samples were processed for measurement of S/P ratio against PRRSV using the same method as described for the purebreds. From now on, we will be referring to S/P ratio following PRRSV vaccination as ***S/P***_***Vx***_. Of these 901 gilts, 811 had farrowing performance recorded for up to 3 parities for litter size traits, including NBA, NSB, MUM, NBD, and TNB. There was no evidence of a PRRSV outbreak during this period. This dataset will hereinafter be referred to as ***P***_***Cross_Vx***_ (performance in vaccinated crossbred sows). Summary statistics for the crossbred/PRRSV vaccination data are presented in Table 1.

### Genotype data

Purebred animals were genotyped using different commercial SNP platforms for 39,610 SNPs. The genotype data were processed according to the breeding company’s pipeline, including the removal of nonsegregating SNP and SNP with poor genotyping scores, and imputation of missing genotypes. Crossbred animals were genotyped for 45,536 using the GGP Porcine HD panel (Neogen GeneSeek, Lincoln, NE) and genotypes with gene call score <0.50, SNP call rate <0.90, and animal call rate <0.90 were removed from the dataset. After quality control and keeping only SNP that overlapped between the purebred and crossbred datasets, 28,579 SNPs were used for subsequent analyses. The SNP calling A/B were translated to nucleotide based on the top (TOP) and bottom (BOT) method by Illumina (llumina SNP Genotyping, 2006). The designations are based on the polymorphism itself, or the contextual surrounding sequence (llumina SNP Genotyping, 2006). Positions of SNP on the genome were based on the *Sus scrofa* 11.1 assembly.

### Statistical analyses

#### Comparison of the purebred and crossbred data

Principal component analysis (PCA) was performed on genotypes to illustrate the overall differences in the genetic makeup between the 2 populations. We also assessed the distribution of *S/P*_*Vx*_ and *S/P_Outbreak_* by plotting the data as histograms and evaluated boxplots for the *P*_*Pure_clean*_, *P*_*Pure_outbreak*_, and *P*_*Cross_Vx*_. For plotting purposes, the data were adjusted for the effects described in Table 2 before plotting.

**Table 2.** Fixed and random effects included in the model for the bivariate analyses

Traits^1^		Fixed effects^2^		Random effects^3^	
Trait 1	Trait 2	Trait 1	Trait 2	Trait 1	Trait 2
*S/P* *_Outbreak_*	*S/P*_*Vx*_	Parity	CG	N/A	N/A
*S/P*_*Vx*_	*P* *_Pure_clean_*:	CG		N/A	
	NBA, NSB, MUM, NBD, TNB		Parity		FMY; PE
	ADG		Birth weight		N/A
	LMD, IMF, BF		Offset weight		N/A
*S/P*_*Vx*_	*P* *_Pure_outbreak_*:	CG	RA; parity	N/A	FMY
	NBA, NSB, MUM, NBD, TNB				
*S/P*_*Outbreak*_	*P*_*Cross_Vx*_:	Parity	Farm; parity	N/A	FMY; PE
	NBA, NSB, MUM, NBD, TNB				

^1^Traits: *S/P_Outbreak_*, antibody response to porcine reproductive and respiratory syndrome (PRRS) virus (PRRSV); *S/P*_*Vx*_, antibody response to PRRSV vaccination; *P*_*Pure_clean,*_ performance of non-infected purebred sows*; P*_*Pure_outbreak*_, performance of PRRSV-infected purebred sows; *P*_*Cross_Vx*_, performance of crossbred PRRSV-vaccinated sows; ADG, average daily gain; LMD, loin muscle depth; BF, backfat; and IMF, intramuscular fat percentage; NBA, number born alive; NSB, number stillborn; MUM, number of piglets mummies; NBD, number born dead; TNB, total number born.

^2^Fixed effects: fixed effects included in the model in addition to the overall intercept. CG, contemporary group (day of blood collection); RA, covariate of 30-d rolling average included to capture the disease progression, following Lewis et al. (2009);

^3^Random effects: random effects included in the model in addition to the animal random effect. FMY, month and year of farrow; PE, permanent environmental effect.

#### Genetic correlations

Previous studies using the same datasets have focused in estimating *r*_g_ between traits within populations. Sanglard et al. (2020) reported *r*_g_ estimates between *S/P*_*Vx*_ and *P*_*Cross_Vx*_, whereas Hickmann et al. (2020) reported *r*_g_ estimates of *S/P_Outbreak_* with *P_Pure_clean_* and *P_Pure_outbreak_*. In order to elucidate the genetic relationship between the crossbred and purebred populations for S/P ratio and reproductive performance, estimates of *r*_g_ were obtained between the two datasets. For that, 4 sets groups of analyses were used to estimate *r*_g_ of traits between the purebred and crossbred datasets, in the presence or not of PRRSV exposure, as listed below:

(1) *S/P*_Outbreak_ and *S/P*_*Vx*_. To obtain *r*_g_ estimates for S/P ratio between PRRSV-infected purebred sows and PRRSV-vaccinated crossbred gilts.

(2) *S/P*_Outbreak_ and *P*_*Cross_Vx*_. To obtain *r*_g_ estimates between S/P ratio in PRRSV-infected purebred sows and reproductive performance in crossbred sows.
(3) *S/P*_*Vx*_ and *P*_*Pure_clean*_. To obtain *r*_g_ estimates between S/P ratio in PRRSV-vaccinated crossbred gilts and reproductive performance in healthy purebred sows.
(4) *S/P*_*Vx*_ and *P*_*Pure_outbreak*_. To obtain *r*_g_ estimates between S/P ratio in PRRSV-vaccinated crossbred gilts and reproductive performance in purebred sows during a PRRSV outbreak.

For these, bivariate Bayesian (BayesC0; Habier et al., 2011) analyses were performed using the following model as described by Cheng et al. (2018a):

$$\begin{array}{l} y_{i}=\mu +X_{i}b+W_{i}u+\sum_{i=1}^{m}z_{ij}\alpha_{j}+e_{j} \end{array}$$ [1]

where **y**_*j*_ is a vector of phenotypes for the 2 traits for individual *i*; **µ** is a vector of overall means for the 2 traits; ***X***_***i***_ is equal $\left[\begin{array}{cc} X_{i1} & 0\\ 0 & X_{i2} \end{array}\right]$, where ***X***_***i*1**_ and ***X***_***i*2**_ are the incidence matrices relating observations to fixed effects for traits 1 and 2 for individual *i*, respectively; ***b*** is equal $\left[\begin{array}{l} b_{1}\\ b_{2} \end{array}\right]$, where **b**_**1**_ and **b**_**2**_ are the vectors of fixed effects for traits 1 and 2, respectively; ***W***_***i***_ is equal $\left[\begin{array}{cc} W_{i1} & 0\\ 0 & W_{i2} \end{array}\right]$, where ***W***_***i*1**_ and ***W***_***i*2**_ are the incidence matrices relating observations to random effects for traits 1 and 2, respectively, for individual *i*; ***u*** is equal $\left[\begin{array}{l} u_{1}\\ u_{2} \end{array}\right]$, where **u**_**1**_ and **u**_**2**_ are the vectors of random effects other than SNP effects; *z*_*ij*_ is the genotype covariate at locus *j* for individual *i* (coded as 0, 1, and 2); *m* is the number of genotyped loci, **α**_*j*_ is the vector of marker effects for locus *j*, where **α**_*j*_ follows a multivariate normal distribution (**MVN**), as **α**_*j*_ ~ MVN (0, ***G***), where $G=\;\left[\begin{array}{cc} \sigma^{2}\beta j_{1} & \sigma \beta j_{1,2}\\ \sigma \beta j_{1,2} & \sigma^{2}\beta j_{2} \end{array}\right]$ and was assumed to have an inverse Wishart prior distribution, $W_{t}^{-1}\left(S_{\beta },\;v_{\beta }\right)$, and **e**_***i***_ is the vector of residuals of *t* traits for individual *i*, where **e**_***i***_ ~ MVN (0, ***R***), where $R=\;\left[\begin{array}{cc} \sigma^{2}e_{1} & 0\\ 0 & \sigma^{2}e_{2} \end{array}\right]$ and was assumed to have an inverse Wishart prior distribution, $W_{t}^{-1}\left(S_{e},\;v_{e}\right)$. Fixed and random effects included for each analysis are in Table 2. For reproductive *P_Pure_clean_* and reproductive *P*_*Vx_cross*_, the model included a random permanent environmental effect to account for the repeated records on the same animal across parities. Bivariate analyses were not performed for the growth and body composition traits during the outbreak due to the low sample size (*n* = 220).

A Markov Chain Monte Carlo with 50,000 iterations was used for each bivariate analysis, with the first 5,000 excluded as burn-in. Estimates of *r*_g_ and their standard errors were obtained as the posterior mean and standard deviation of the correlation between the sampled genomic breeding values (**GBVs**) for the 2 traits at each iteration. The posterior probability (*P*_0_) of *r*_g_ being greater (if the posterior *r*_g_ was >0) or less than zero (if the posterior *r*_g_ was <0) was tested and considered significant when *P*_0_ > 0.90. All analyses were performed in the *JWAS* package (Cheng et al. (2018b), from Julia software (Bezanson et al., 2015).

#### Bivariate genome-wide association studies (BiGWAS)

To identify Quantitative Trait Loci (QTL), BiGWAS using BayesB (Habier et al., 2011) was performed for all pairs of traits, using the model of equation 1, except that in the BayesB method, each SNP had prior probabilities of being fitted for only one of the traits, for both traits, or for none of the traits in each iteration. For all analyses, we defined a probability of 0.10 for the SNPs to have an effect on both traits simultaneously, 0.05 to have an effect in only one of the traits, and 0.80 to not have any effect. For each trait, a 1-Mb window with a posterior probability of inclusion (**PPI**) >0.70 (Garrick and Fernando, 2013) was deemed to be contain QTL. The analyses were performed using the *JWAS* package (Cheng et al. (2018b). Linkage disequilibrium (**LD**) between SNP within QTL regions was estimated as *r*^*2*^ using Plink (Purcell et al., 2007) and plotted using Haploview (Barrett et al., 2005).

#### Genetic covariances across the genome

 To identify the regions of the genome explaining the genetic covariance between 2 traits for all 4 groups of traits described for the genetic correlation, we estimated the proportion of the genetic covariance between the 2 traits explained by sliding regions across the genome. Analyses were performed using BayesA and BayesB (Habier et al., 2011) with the model presented in equation 1. In BayesA, all SNPs are simultaneously fitted in the model, whereas in BayesB, the same proportion of SNPs being fitted in the model described for the BiGWAS were used in this analysis. Analyses were performed with both methods for complementarity. For instance, BayesA was used to represent the infinitesimal model allowing for large QTL, such as the one for S/P ratio in SSC 7 (Serão et al., 2014, 2016; Hickmann et al., 2020; Sanglard et al., 2020). However, BayesB was also used to better represent the oligogenic genomic architecture of S/P ratio.

The contribution of genomic regions to the total genetic covariances between traits were estimated using sliding windows of 10 SNPs, moving by 2 SNPs at a time. For each trait, the sampled GBV of each individual was calculated by multiplying the SNP genotypes (***g***) of each individual by the sampled marker effects (***m***) of 10 SNPs for each iteration of the MCMC:

$$\begin{array}{l} sampled\;GBV={\left[\begin{array}{ccc} g_{1,1} & \cdots & g_{1,10}\\ \vdots & \ddots & \vdots \\ g_{906,1} & \cdots & \;\;\;g_{906,10} \end{array}\right]}_{\#individual\;x\;\#SNP}\\ {}*\;\;\;{\left[\begin{array}{ccc} m_{1,1} & \cdots & m_{10,1}\\ \vdots & \ddots & \vdots \\ m_{10,1} & \cdots & m_{10,450} \end{array}\right]}_{\#SNP\;x\;\#iteration}, \end{array}$$

resulting in a matrix of the sampled $GBV_{\#individual\;x\;\#iteration}$ for each trait. Then, the covariance between the sampled GBV of individuals between the 2 traits was calculated for each iteration, resulting in a vector of sampled covariances: $COV^{\prime }={\left[cov_{1}\;\ldots \;cov_{450}\;\right]}_{\#interaction}$. The proportion of covariance explained by each window was calculated by dividing COV by the total covariance across the genome (covariance fitting all the markers) for each iteration. Finally, the posterior proportion covariance was calculated as the average of the proportion covariances across iterations. The posterior probability (*P*_0_) of the proportion covariance to be greater or smaller than the expected absolute proportion explained by 10 SNPs (i.e., 10/28,579 SNPs = 0.00035) was calculated for each sliding window and results are been shown for *P*_0_ ≥ 0.90. Positive proportions in the plot refer to the regions contributing for a positive covariance between 2 traits while negative proportions in the plot refer to the regions contributing for a negative covariance between 2 traits. The sign of the proportions represented the sign of the posterior covariance between the 2 traits analyzed. Additionally, we investigated the genes included in identified regions in the BiGWAS and genetic covariance analyses to identify candidate genes with functions associated with immune response and reproductive performance.

#### Effect of major SNP on antibody response and performance traits

We tested the effect of the SNP that were previously identified in the univariate GWAS for *S/P_Outbreak_* (ASGA0032063) and *S/P*_*Vx*_ (H3GA0020505; Sanglard et al., 2020) for all traits (S/P ratio and performance) evaluated in this study. For that, the genotypes for these 2 SNPs were fitted as categorical fixed effects in a univariate version of the model equation 1, along with all other effects described in Table 2. For each SNP, estimates of the additive and dominance effects were calculated using orthogonal contrasts. The 2 SNPs were fitted using 2 strategies: 1 SNP at a time or both simultaneously. This was done because of their proximity (0.8 Mb apart), which could result in these 2 SNPs capturing the same QTL. The *P*_0_ for the additive and dominance effects to be greater (when the posterior additive or dominance effect was greater than zero) or less (when the posterior additive or dominance effect was less than zero) than zero were tested and considered significant when *P*_0_ > 0.90.

## Results

### Overview of the 2 populations

**Figure 2.**
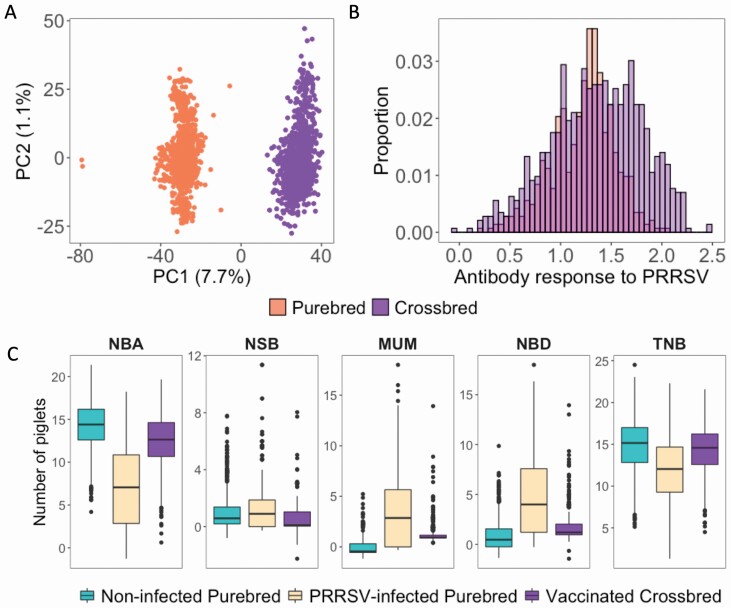
Comparison of genotypic and phenotypic data between purebred and commercial crossbred animals. (A) PC analysis of the genotypes for purebred (coral) and crossbred (purple) sows. X- and y-axis correspond to PC1 and PC2, respectively, with the percentage of the variation explained by the respective PCs in parenthesis. (B) Histogram of distribution of the data for antibody response measured as sample-to-positive (S/P) ratio to PRRSV outbreak (purebred; coral) and vaccination (crossbred; purple). (C) Box-plot of the reproductive performance data, including NBA, NSB, MUM, TNB, and NBD, for non-infected purebred sows (green), PRRSV-infected purebred sows (coral), and non-infected crossbred commercial sows (purple). S/P ratio data and reproductive performance were adjusted for fixed and random effects (Table 2).

We performed PCA on genotypes from the 2 populations (purebred and crossbred; Figure 2A) to investigate the individuals’ genetic background. Principal components (**PC**) 1 (**PC1**) and 2 (**PC2**) explained 7.7% and 1.1% of the variation in the genotypes, respectively. PC1 separated the 2 populations, while PC2 did not. Each population formed a single cluster, without connections between the crossbred and purebred animals.

The raw mean ± standard deviation for *S/P_Outbreak_* was 1.41 ± 0.45, and for *S/P*_*Vx*_ was 1.22 ± 0.31, with both distributions having a normal distribution (Figure 2B). Based on the threshold of S/P ≥ 0.4, 538 and 891 animals were positive for PRRSV after outbreak and vaccination, respectively. We can observe that some animals were considered negative based on this threshold diagnostic even though we know they were vaccinated. Several studies have reported moderate to high heritability estimates of S/P ratio when this trait was analyzed as a quantitative continuous variable following a normal distribution, indicating that there is a strong relationship between phenotypic and genetic values for this trait (Serão et al., 2014, 2016; Abella et al., 2019; Sanglard et al., 2020). As seen in Figure 2B, the S/P ratio data used in this study followed a normal distribution. In fact, all crossbred animals were vaccinated for PRRSV, indicating that PRRSV-negative animals (i.e., with S/P < 0.4) have low antibody levels to PRRSV. Hence, S/P ratio was analyzed as a continuous variable in this study. The distributions of the adjusted *P_Pure_clean_* and *P*_*Cross_Vx*_ data are shown in Figure 2C. In general, the *P_Pure_outbreak_* data had smaller litter size and greater litter mortality than the *P_Pure_clean_* and *P*_*Cross_Vx*_ data. The variability of the *P*_*Pure_outbreak*_ data was also higher than for the *P_Pure_clean_* and *P*_*Cross_Vx*_ data.

### Genetic correlations

Estimates of *r*_g_ are shown in Table 3. The estimate of *r*_g_ of *S/P*_*Vx*_ with *S/P*_Outbreak_ was high, with 0.72 ± 0.18 (*P*_0_ = 1.00) and 95% credible interval of [0.26; 0.92]. Estimates of *r*_g_ between *S/P*_*Vx*_ and *P_Pure_clean_* traits were significant for NBA (0.50 ± 0.23; *P*_0_ = 0.95), BF (−0.47 ± 0.18; *P*_0_ = 0.99), and IMF (0.83 ± 0.08; *P*_0_ = 0.95). Estimates of *r*_g_ of *S/P*_*Vx*_ with reproductive P*_Pure_outbreak_* traits were overall low and mostly negative, with significant estimates for MUM (−0.37 ± 0.21; *P*_0_ = 0.95) and TNB (−0.29 ± 0.18; *P*_0_ = 0.94). In contrast, estimates of *r*_g_ of *S/P_Outbreak_* with reproductive *P*_*Cross_Vx*_ traits were, in general, positive and low, with a significant *r*_g_ for TNB (0.30 ± 0.20; P_0_ = 0.92). Overall, the strength of the genetic relationship between S/P ratio and reproductive performance varied depending on the population but, in most of the scenarios, the *r*_g_ was positive with litter size traits and negative with piglet mortality traits.

**Table 3.** Genetic correlations between antibody response and performance

Trait 1	Trait 2	Genetic correlation	*P*_0_
*S/P* *_Outbreak_*	*S/P Vx*	0.72 (0.18)	1.00
*S/P*_*Vx*_	*P*_*Pure_clean*_		
	ADG	0.09 (0.36)	0.56
	LMD	0.06 (0.17)	0.65
	IMF	0.83 (0.08)	1.00
	BF	−0.47 (0.18)	0.99
	NBA	0.50 (0.23)	0.95
	NSB	0.00 (0.23)	0.50
	MUM	−0.02 (0.23)	0.47
	NBD	−0.11 (0.23)	0.70
	TNB	0.27 (0.37)	0.75
*S/P*_*Vx*_	*P*_*Pure_outbreak*_		
	NBA	0.07 (0.22)	0.49
	NSB	0.05 (0.19)	0.60
	MUM	−0.38 (0.21)	0.95
	NBD	−0.06 (0.16)	0.68
	TNB	−0.30 (0.18)	0.94
*S/P*_*Outbreak*_	*P*_*Cross_Vx*_		
	NBA	0.23 (0.25)	0.82
	NSB	0.05 (0.23)	0.60
	MUM	0.05 (0.26)	0.54
	NBD	0.16 (0.24)	0.76
	TNB	0.30 (0.20)	0.92

Traits: *S/P*_*Outbreak*_, antibody response to porcine reproductive and respiratory syndrome (PRRS) virus outbreak; *S/P*_*Vx*_, antibody response to PRRS virus (PRRSV) vaccination; *P*_*Pure_clean,*_ performance of non-infected purebred sows*; P*_*Pure_clean*_, performance of non-infected purebred sows; *P*_*Pure_outbreak*_, performance of PRRSV-infected purebred sows; *P*_*Cross_Vx*_, performance of crossbred PRRSV-vaccinated sows; ADG, average daily gain; LMD, loin muscle depth; BF, backfat; and IMF, intramuscular fat percentage; NBA, number born alive; NSB, number stillborn; MUM, number of piglets mummied; NBD, number born dead; TNB, total number born.

*P*_0_: posterior probability of the genetic correlation estimates being greater or less than zero.

### Bivariate genome-wide association studies

Only 2 QTL were identified in the BiGWAS performed for pairs of traits (Table 4). For the analysis of *S/P_Outbreak_* and *S/P*_*Vx*_, a QTL on *Sus scrofa* chromosome (**SSC**) 7 (25 to 26Mb) explained 19.8 (PPI = 1.00) and 25.6% (PPI = 1.00) of the total genetic variance explained by the markers (**TGVM**) for *S/P_Outbreak_* and *S/P*_*Vx*_, respectively. For these QTL, most of the TGVM was explained by the H3GA0020505 SNP, which explained 19.6% (PPI = 1.00) and 21.0% (PPI = 1.00) of the TGVM for *S/P_Outbreak_* and *S/P*_*Vx*_, respectively.

**Table 4.** Significant^1^ QTL from the bivariate GWAS

Trait 1^2^	Trait 2^2^	SSC^3^	Window start (Kb)	Window end (Kb)	#SNP^4^	Main SNP^5^	Trait 1		Trait 2	
							% of TGVM^6^	PPI^1^	% of TGVM^6^	PPI^1^
*S/P*_Outbreak_	*S/P*_*Vx*_	7	25003013	25967157	10	H3GA0020505	19.8	1.00	25.6	1.00
*S/P*_*Vx*_	IMF	7	24217931	24865378	5	SIRI0000155	6.5	0.86	5.5	0.77

^1^Significant QTL were considered when PPI was >0.70 for both traits.

^2^Traits: *S/P*_Outbreak_, antibody response to porcine reproductive and respiratory syndrome (PRRS) virus (PRRSV); *S/P*_*Vx*_, antibody response to PRRSV vaccination; IMF, intramuscular fat.

^3^SSC: *Sus scrofa* chromosome.

^4^#SNPs: number of SNPs within the window.

^5^Main SNP: SNP within a window explaining most of the genetic variance.

^6^TGVM: total genetic variance explained by the markers.

For the analysis of *S/P*_*Vx*_ and IMF, a QTL on SSC 7 (24.2 to 24.8 Mb) explained 6.5 (PPI = 0.86) and 5.5% (PPI = 0.77) of the TGVM for *S/P*_*Vx*_ and IMF, respectively, with the SIRI0000155 SNP explaining 6.4% (PPI = 0.86) and 5.5% (PPI = 0.77) for *S/P*_*Vx*_ and IMF, respectively. For the analyses of S/P ratio and reproductive performance traits, the same QTL on SSC 7 (25 to 26Mb) was identified (PPI > 0.7) for S/P ratio but this QTL had no significant effect on reproductive traits (Supplementary Table 1).

### Genetic covariances of sliding windows

**Figure 3.**
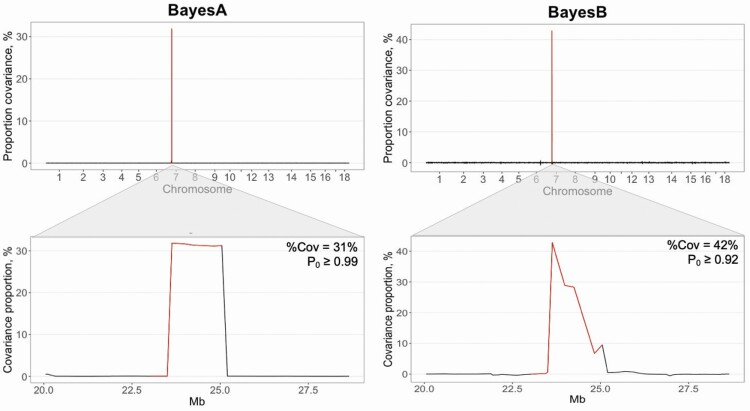
Genetic covariance of sample-to-positive ratio to PRRSV following a natural outbreak and following vaccination with a modified live virus using BayesA and BayesB methods. The proportion of covariance was estimated for sliding windows of 10 SNPs moving each 2 SNPs. *P*_0_ corresponds to the lowest posterior probability of the proportion covariance to be greater or smaller than the expected absolute proportion explained by 10 SNPs (i.e., 10/28,579 SNPs = 0.00035), and %Cov corresponds to the average genetic covariance explained by the SNP windows located on the major histocompatibility complex region (SSC 7; ~23 to 26 Mb), which is highlighted in red.

Genetic covariances between *S/P*_*Vx*_ and *S/P_Outbreak_* across the genome are shown in Figure 3. The genomic region on SSC 7 (23.6 to 25.9 Mb; *P*_0_ ≥ 0.92) explained ~31% and 42% of the genetic covariance (**%Cov**) between these 2 traits when using BayesA and BayesB, respectively. This region is known as the MHC region, which can be further classified as MHC class I (~22.5 to 23.6 Mb), MHC class II (~24.8 to 25.3 Mb), and MHC class III (~23.6 to 24.2 Mb) (Hammer et al., 2020).

Results for the covariance between S/P ratio and reproductive performance are presented in Supplementary Figure 1 (BayesA) and 2 (BayesB). The same genomic window on SSC 7 (23.6 to 25.9 Mb) was associated (*P*_0_ ≥ 0.90) with the genetic covariance between *S/P*_*Vx*_ with IMF (%Cov = 18%) and TNB (%Cov = 25%) *P_Pure_clean_*. Also, 10-SNP rolling windows in this region were associated (*P*_0_ ≥ 0.92) with the genetic covariance between *S/P*_*Vx*_ with TNB (%Cov = 22%) *P_Pure_outbreak._* There were no genomic regions significantly (*P*_0_ ≤ 0.77) associated with the genetic covariance between *S/P_Outbreak_* and *P*_*Cross_Vx*_. This is in accordance with the overall low *r*_g_ estimates between *S/P_Outbreak_* and *P*_*Cross_Vx*_.

Overall, regions on SSC 7 inside the MHC region play an important role in explaining a substantial proportion of the genetic covariances between S/P ratio to PRRSV vaccination (i.e., and *S/P*_*Vx*_) with reproductive performance in purebred sows (i.e., *P_Pure_clean_* and *P_Pure_outbreak_*).

### Effects of major SNP on antibody response and performance traits

**Figure 4.**
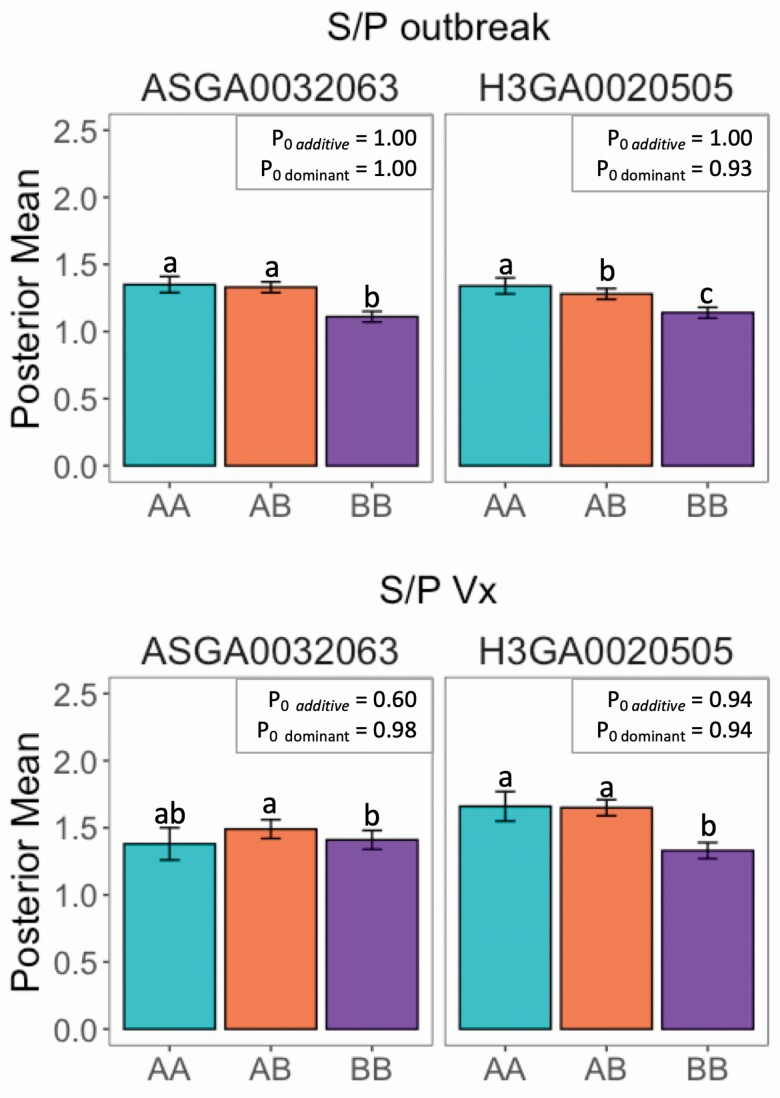
Effect of SNP ASGA0032063 and H3GA0020505 on antibody response, measured as sample-to-positive (S/P) ratio, to PRRS virus outbreak (*S/P_Outbreak_*) and PRRS vaccination (*S/P*_*Vx*_). The posterior probabilities (*P*_0_) for the additive and dominance effects of the SNP to be different than zero. Each color corresponds to 1 SNP genotype. Error bars represent the posterior standard deviation of the mean genotype across iterations. Different letters represent significant difference between the genotypes at *P*_0_ > 0.90.

The effects of the ASGA0032063 and H3GA0020505 SNP were estimated for all traits. Posterior probabilities of additive and dominance effects of being greater than zero are shown in Table 5, while posterior means, posterior standard deviation, and posterior probabilities of additive and dominance effects are in Supplementary Table 2. Posterior means of *S/P_Outbreak_* and *S/P*_*Vx*_ for each SNP (fitting one at a time) are presented in Figure 4. When fitting each SNP separately, the dominant effects of both SNPs were significant (*P*_0_ ≥ 0.93) for *S/P_Outbreak_* and *S/P*_*Vx*_. The posterior means of *S/P_Outbreak_* for ASGA0032063 showed a complete dominance mode-of-action for its genotypes, with AA = AC > CC, whereas for H3GA0020505 genotypes, a partial dominance mode-of-action was observed, with AA > AC > CC (Figure 4). The posterior means of *S/P*_*Vx*_ for ASGA0032063 has a complex relationship, with AC > CC and both not differing from AA, whereas for H3GA0020505, there was a complete dominance mode-of-action, with AA = AC > CC (Figure 4). When both SNPs were fitted in the model simultaneously, H3GA0020505 was not significantly associated (*P*_0_ ≤ 0.73) with *S/P_Outbreak_* (Table 5). Also, the mode-of-action of ASGA0032063 on *S/P*_*Vx*_ was slightly different than when both SNPs were fitted separately, showing a significant (*P*_0_ = 1.00) additive effect (Table 5), with AA < AC = CC.

**Table 5.** Posterior probabilities^1^ of additive (ADD) and dominance (DOM) effects to be different than zero for the SNP H3GA0020505 and ASGA0032063 for all traits

	Fitting SNP simultaneously				Fitting one SNP at a time			
Trait^2^	H3GA0020505		ASGA0032063		H3GA0020505		ASGA0032063	
	ADD	DOM	ADD	DOM	ADD	DOM	ADD	DOM
*S/P* *_Outbreak_*	0.73	0.69	**1.00**	**1.00**	**1.00**	**0.93**	**1.00**	**1.00**
*S/P*_*Vx*_	**1.00**	**1.00**	**1.00**	**1.00**	**0.94**	**0.94**	0.60	**0.98**
*P*_*Pure_clean*_								
ADG, kg/d	0.67	**0.90**	0.88	**0.99**	0.86	0.60	**0.97**	**0.97**
LMD, cm	0.83	0.82	**1.00**	0.53	0.85	**0.93**	**0.92**	0.75
IMF, %	**0.92**	0.83	0.72	0.79	0.80	0.76	0.80	0.66
BF, cm	0.55	0.68	0.54	**0.94**	0.59	**0.98**	0.84	0.81
NBA	0.80	0.58	0.69	0.52	0.74	0.57	0.53	0.51
NSB	0.84	0.74	0.85	0.87	0.66	0.53	**1.00**	**1.00**
MUM	**0.91**	0.50	**1.00**	**1.00**	**0.92**	0.66	0.78	0.84
NBD	**0.91**	0.69	0.82	0.68	0.81	0.63	0.60	0.62
TNB	**0.98**	0.75	0.86	0.67	**0.94**	0.74	0.52	0.53
*P*_*Pure_outbreak*_								
ADG, kg/d	0.53	0.88	0.62	0.89	0.72	0.70	0.59	0.86
LMD, cm	0.67	0.78	0.53	**0.98**	0.72	0.56	0.75	0.56
IMF, %	0.74	0.74	0.54	0.89	0.66	0.88	0.71	**0.97**
BF, cm	**1.00**	**1.00**	0.60	0.84	0.84	0.65	0.67	**0.95**
NBA	**0.93**	0.84	0.77	0.79	**0.92**	0.72	0.63	0.60
NSB	0.57	0.90	0.61	0.81	0.52	0.80	0.63	0.57
MUM	0.67	0.76	0.70	0.65	0.88	0.88	0.89	0.79
NBD	0.72	0.63	0.68	0.72	**0.92**	0.52	0.88	0.71
TNB	**0.93**	0.51	**0.98**	0.77	0.50	0.71	**0.93**	0.81
*P*_*Cross_Vx*_								
NBA	**0.96**	0.88	**0.90**	**0.95**	**0.97**	0.87	0.89	**0.97**
NSB	0.80	0.51	0.81	0.77	0.74	0.54	0.80	0.78
MUM	**0.91**	0.76	0.54	**1.00**	0.87	0.70	0.52	0.72
NBD	0.61	0.71	0.80	0.70	0.68	0.68	0.80	0.71
TNB	**0.99**	**0.96**	0.54	**1.00**	**0.99**	**0.95**	0.76	**0.92**

^1^Significant associations (*P*_0_ ≥ 0.90) in bold.

^2^Traits: *S/P_Outbreak_*, antibody response to porcine reproductive and respiratory syndrome (PRRS) virus (PRRSV); *S/P*_*Vx*_, antibody response to PRRSV vaccination; *P*_*Pure_clean*_, performance of non-infected purebred sows*; P*_*Pure_clean*_, performance of non-infected purebred sows; *P*_*Pure_outbreak*_, performance of PRRSV-infected purebred sows; *P*_*Cross_Vx*_, performance of crossbred PRRSV-vaccinated sows; ADG, average daily gain; LMD, loin muscle depth; BF, backfat; and IMF, intramuscular fat percentage; NBA, number born alive; NSB, number stillborn; MUM, number of piglets mummied; NBD, number born dead; TNB, total number born.

For performance traits, several associations were found (*P*_0_ ≥ 0.90) for these 2 SNPs. For reproductive *P_Pure_clean_* traits, H3GA0020505 had an additive effect (*P*_0_ ≥ 0.91) on MUM, NBD, and TNB. For MUM, ASGA003206 also had a dominance effect (*P*_0_ = 1.00) when fitting both SNPs simultaneously. For reproductive *P_Pure_outbreak_* traits, H3GA0020505 had an additive effect (*P*_0_ ≥ 0.92) on NBA and TNB. ASGA003206 had an additive effect (*P*_0_ = 0.98) on TNB. For reproductive *P*_*Cross_Vx*_, H3GA0020505 had an additive effect (*P*_0_ ≥ 0.91), and ASGA003206 had a dominance effect (*P*_0_ ≥ 0.95) on NBA, MUM, and TNB.

For body composition and growth traits in *P_Pure_clean_*, both SNPs had significant dominance effects (*P*_0_ ≥ 0.90) for ADG, ASGA0032063 had an additive effect (*P*_0_ = 1.00) on LMD. H3GA0020505 had an additive effect (*P*_0_ = 0.92) on IMF. ASGA0032063 had a dominance effect (*P*_0_ = 0.94) on BF. For *P_Pure_outbreak_*, ASGA0032063 and H3GA0020505 had a dominance effect (*P*_0_ ≥ 0.98) on LMD and BF, respectively.

In general, genotypes AA and AC of the H3GA0020505 SNP were associated with higher *S/P*_*Vx*_ and *S/P_Outbreak_* than CC. For ASGA0032063_,_ genotypes AA and AC had higher *S/P_Outbreak_* than CC, but the AC genotype for both SNPs was associated with higher *S/P*_*Vx*_ than CC. For reproductive performance, genotypes AA and AC of the H3GA0020505 SNP were associated with greater litter size traits than CC genotypes but also higher litter mortality, with the AC genotype being associated with the best overall performance for both populations. For the ASGA0032063 SNP, in general, the CC genotype was associated with larger litter size and lower litter mortality than genotype AA, whereas genotype AC also associated with overall better performance for all traits.

## Discussion

Previous studies using the same datasets used in the current study have reported genetic correlation estimates of antibody response to PRRSV with reproductive performance using the purebred (Hickmann et al., 2020) and crossbred (Sanglard et al., 2020) datasets. In summary, Hickmann et al. (2020), using purebred sows during a PRRSV outbreak, reported favorable positive estimates of *r*_g_ of *S/P_Outbreak_* with TNB and NBA (*r*_g_ ≥ 0.54), and negative low estimates with piglet mortality traits (*r*_g_ ≤ 0.12). This correlation of *S/P_Outbreak_* with reproductive performance was also favorable in non-infected purebred sows prior to the outbreak, with *r*_g_ = 0.17 (NBA) and *r*_g_ ≤ −0.33 (NBD and NSB). Sanglard et al. (2020), using PRRSV-vaccinated crossbred animals, reported favorable estimates of *r*_g_ of *S/P*_*Vx*_ with subsequent reproductive performance, such as *r*_g_ = 0.61 (0.16) for NBA at first parity and *r*_g_ = −0.84 (0.05) for NSB at third parity. Given these favorable results, in the present study, we assessed the genetic relationship of S/P ratio and performance between the two datasets. In other words, we estimated the *r*_g_ of S/P ratio (outbreak and vaccinated) from 1 of the 2 datasets with performance from the other dataset.

Animals used in this study were from maternal lines. Hence, there is less emphasis in the selection for growth in these animals, since these maternal lines are mainly selected for improved reproductive performance. Although it is possible to measure growth traits of interest in their offspring, these data were not available for analyses. On a side note, only crossbred sows that had been PRRSV-vaccinated had genomic information available and were used in the study. Vaccination of the F1 animals was performed right after gilts entered the commercial farms, around 160 d before insemination. After PRRSV vaccination, total PRRSV-specific antibody response may last in the blood of animals for up to ~180 d (Andraud et al., 2018). Thus, at the time of first farrowing (~160 d after vaccination), it is expected that the presence of antibody level in the blood of these animals was minimum, if any. In addition, the estimate of *r*_g_ of *S/P*_*Vx*_ with reproductive performance of non-infected purebred sows was positive and favorable with NBA (0.50) and TNB (0.27) and negative and favorable with NBD (−0.11). Thus, we expected a similar favorable *r*_g_ of *S/P*_*Vx*_ with reproductive performance in non-vaccinated crossbred sows. Presumably, the estimate of *r*_g_ of *S/P*_*Vx*_ with reproductive performance of non-vaccinated crossbred sows would be similar to the estimate for PRRSV-vaccinated crossbred sows in this study. Future studies are warranted to obtain these estimates for nonvaccinated commercial sows based on the S/P ratio information measured in genetically related commercial sows vaccinated for PRRSV.

Most studies on genomics of S/P ratio in sows after a PRRSV outbreak have used purebred populations (Serão et al., 2014; Putz et al., 2019; Hickmann et al., 2020). Although genetic selection is performed in purebred herds in the nucleus, the targeted trait of interest is to be improved is performance of crossbred individuals in the commercial level. Thus, it would be interesting to obtain the estimates of *r*_g_ between S/P ratio and reproductive performance in commercial crossbred animals during a PRRSV outbreak. Although PRRSV outbreaks are more common at the commercial level than in the nucleus, the logistics for data collection at commercial-level farms is generally challenging. Unfortunately, such source of data was not available for this study.

Finally, PRRSV vaccination is not performed in purebred individuals in nucleus herds. This happens to avoid vaccination having an impact in the genetic evaluation of the population and issues with selling semen and animals being tested positive due to vaccination. On the other hand, PRRSV vaccination is a common practice used by producers at the commercial level to reduce impact of the PRRSV on animal performance. Thus, it is expected that S/P ratio data due to PRRSV vaccination should be only generated and collected in crossbred commercial animals. Therefore, the use of PRRSV vaccination in purebred nucleus animals might not be a practical strategy to generate S/P data for selection purposes.

### Genetic correlations

The estimate of *r*_g_ of *S/P*_*Vx*_ with *S/P_Outbreak_* was <1 (0.72; 95% credible interval = [0.26 to 0.92]). This moderate-to-high correlation suggests that these 2 traits are under similar genetic control; however, may not be the same trait. Nonetheless, previous studies have shown similarities between S/P ratio to PRRSV in PRRSV-outbreak and in PRRSV-vaccinated pigs at the genomic level. Serão et al. identified 2 major QTL on SSC 7 that combined explained over 30% of the TGVM of S/P ratio in purebred sows during a PRRSV outbreak (Serão et al., 2014) and in crossbred gilts after acclimation (Serão et al., 2016). One of these QTL is located in the MHC. Sanglard et al. (2020), using the same animals used in this study, showed that the MHC QTL is also associated with *S/P*_*Vx*_ in PRRSV-vaccinated crossbred gilts. These results support that *S/P*_*Vx*_ with *S/P_Outbreak_* could be under similar genetic control.

The estimate of *r*_g_ between *S/P*_*Vx*_ and *S/P_Outbreak_* obtained was <1 but was inside the range (0.70 to 0.90) for the *r*_g_ observed between purebred and crossbred performances of most traits in pigs (Mulder et al., 2016; Wientjes and Calus, 2017). Three main factors may be playing a role in this *r*_g_ is being smaller than one: the difference in the immune response to vaccination and outbreak, genotype-by-environment interaction, and non-additive genetic effects. First, although some differences can be observed on the innate immune response (i.e., PRRSV vaccination does not stimulate IL-10 as observed in wild-type infections; Balasch et al., 2019), vaccination and wild-type infections stimulate similar acquired immune responses. For example, in both cases, there is a delay in the antibody response to this pathogen (Montaner-Tarbes et al., 2019). Second, the traits were collected in two different environments: *S/P_Outbreak_* in the nucleus and *S/P*_*Vx*_ at the commercial level. The phenomenon of genotype-by-environment interaction is especially important for immune-related traits in the swine industry. Management is expected to be different between the 2 environments, including diet and handling. The estimate of residual variance was almost twice as high for *S/P*_*Vx*_ than for *S/P_Outbreak_*, corroborating the differences expected between the two environments. Third, we have different populations in each environment, purebred (nucleus) and crossbred animals (commercial), which could result in different genetic effects impacting the expression of the traits between populations, such as non-additive genetic effects (i.e., dominance and epistastic effects). Crossbred populations are expected to have more heterozygotes loci across the genome and may have different allelic frequencies than parental purebred lines. Thus, the estimated allele substitution effect of the SNP can be different between the 2 populations.

Previous studies have reported that S/P ratio is highly and favorably correlated with reproductive performance during a PRRSV outbreak (Serão et al., 2014; Putz et al., 2019; Hickmann et al., 2020). In our study, we estimated the *r*_g_ of *S/P_Outbreak_* with reproductive *P*_*Cross_Vx*_ and obtained moderate and favorable estimate (0.30) with TNB. However, estimates of *r*_g_ with litter mortality traits (NSB, MUM, and NBD) were low and not significant. There are no reports in the literature for *r*_g_ estimates of *S/P_Outbreak_* with reproductive *P*_*Cross_Vx*_ by which to compare our results. Thus, we estimated the expected *r*_g_ of *S/P_Outbreak_* with TNB *P*_*Cross_Vx*_ by multiplying the *r*_g_ estimate of *S/P_Outbreak_* with TNB *P_Pure_outbreak_* (0.54; Hickmann et al., 2020) by the *r*_g_ estimate of NBA *P_Pure_outbreak_* with NBA *P*_*Cross_Vx*_ (0.82; data not shown) and obtained an expected *r*_g_ = 0.44. Although the expected *r*_g_ was greater than the observed (*r*_g_ = 0.30), it is important to note that the expected *r*_g_ assumes that these 2 events are independent of each other. Thus, the expected *r*_g_ based on this calculation may be overestimated. Additionally, we obtained a favorable estimate of *r*_g_ of *S/P*_*Vx*_ with *P_Pure_clean_*. The estimate was positive and moderate (*r*_g_ ≥ 0.50) with NBA while not significant with piglet mortality traits (−0.11 ≤ *r*_g_ ≤ 0).

Surprisingly, the estimate of *r*_g_ of *S/P*_*Vx*_ with TNB *P_Pure_outbreak_* was negative and moderate (−0.30). This could be due to the moderate negative correlation of *S/P*_*Vx*_ with MUM (−0.38), since the estimate of *r*_g_*S/P*_*Vx*_ with NBA was very low (0.06) and not significant. There are no reports in the literature of *r*_g_ of *S/P*_*Vx*_ with traits in PRRSV-infected purebred animals. Using the same data used in this study, Sanglard et al. (2020) showed that *S/P*_*Vx*_ is favorably genetically correlated with NBA (0.61) at parity 1 and NSB (−0.84) and MUM (−0.83) at parity 3 in commercial crossbred sows previously vaccinated for PRRSV. By combining these 2 results, we expect that selection for increased *S/P*_*Vx*_ collected at the commercial level to have a favorable impact on litter size and litter mortality, not only for crossbred sows but also for non-infected and PRRSV-infected purebred animals in the nucleus.

We had initially expected that *S/P*_*Vx*_ and *P_Pure_outbreak_* to have stronger *r*_g_ based on the *r*_g_ estimates *S/P*_*Vx*_ with *P_Pure_clean_* in this study and *S/P*_*Vx*_ with *P*_*Cross_Vx*_ in Sanglard et al. (2020). Results from our study suggest that the *r*_g_ of *S/P*_*Vx*_ with reproductive performance is stronger under a clean condition than under a PRRSV outbreak. For example, the *r*_g_ estimates between *S/P*_*Vx*_ with *P*_*Pure_clean*_ were, in general, stronger than with *P_Pure_outbreak_*. One point to consider is the health status of purebred animals in the nucleus. Vaccination to PRRSV was not performed in the purebred animals used in this study. However, they received other types of vaccination, such as porcine circovirus type 2 (**PCV2**) and *E. coli*. Although the nucleus is considered a “clean” environment, the immune system of these animals has been already stimulated. In fact, Dunkelberger et al. (2017) reported a high *r*_g_ estimate (>0.90) between viral loads of PRRSV and PCV2 in nursery pigs previously vaccinated for PRRSV and then co-infected with PRRSV and PCV2. Hence, the stronger *r*_g_ estimates obtained between *S/P*_*Vx*_ with *P_Pure_clean_* compared with *S/P*_*Vx*_ with *P_Pure_outbreak_* could be explained, at least in part, due to the previously stimulus of the immune system in both the crossbred and purebred (before the PRRSV outbreak) populations.

The estimates of *r*_g_ of *S/P*_*Vx*_ with body composition traits in non-infected purebred animals were high and favorable for BF (−0.47) and for IMF (0.83). These results were unexpected at first because the estimate of *r*_g_ of BF with IMF is expected to be moderate and positive (Lo et al., 1992; Rozycka et al., 1998; Hernández-Sánchez et al., 2013). With this, we would expect the estimates of *r*_g_ of both traits with *S/P*_*Vx*_ to have the same direction. The estimate of *r*_g_ of BF with IMF in our population was, however, positive and low (0.25 ± 0.13), which may explain their respective estimates of *r*_g_ with *S/P*_*Vx*_. Meeker et al. (1987) reported no genetic correlation between BF thickness and antibody response to pseudorabies virus vaccination in Landrace and Yorkshire piglets at 28 d of age. Hess et al. (2018) also investigated the *r*_g_ of S/P ratio measured in nursery pigs at 21 d of age following experimental infection with PRRSV with growth rate following infection and found negative estimates of *r*_g_ with early growth after infection but positive estimates later on. In our study, the estimate of *r*_g_ of *S/P*_*Vx*_ with ADG from birth to offtest was low (0.06). These results suggest that selection for increased *S/P*_*Vx*_ would have a favorable impact of body composition traits in non-infected purebred sows, with a decrease in BF and increase in marbling, increasing the value of the body composition.

Altogether, the results obtained in our study suggest that selection for S/P ratio after a PRRSV outbreak or vaccination would yield a favorable impact on the reproductive performance and body composition traits of non-infected purebred, PRRSV-exposed purebred, and crossbred sows.

### BiGWAS and genetic covariances across the genome

The BiGWAS for *S/P_Outbreak_* with *S/P*_*Vx*_ revealed the region on SSC 7 (~25 to 26 Mb) explaining most of the total genetic covariance explained by the markers. This region overlaps with the region explaining ~33% of the genetic covariance between these traits on SSC 7 (23.6 to 25.9 Mb) based on the analyses of genetic covariance for sliding windows and the previously region identified in the univariate GWAS using the same dataset (Sanglard et al., 2020). This region includes the H3GA0020505 SNP, which explained most of the TGVM (~30%) for *S/P*_*Vx*_ in the univariate GWAS (Sanglard et al., 2020). This region also embraces the MHC class II and extended class II, where several genes associated with immune response are located. An extensive review of candidate genes for S/P ratio can be seen by Sanglard et al. (2020). These results corroborate that genes located in the MHC class II are strong candidate to be associated with S/P ratio to PRRSV outbreak and vaccination in purebred and crossbred sows.

The significant region on SSC 7 (24.2 to 24.8 Mb) for *S/P*_*Vx*_ and IMF also overlaps with the region explaining the genetic covariance between these 2 traits on SSC 7 (23.6 to 25.9 Mb). This region is located within the MHC class II and a potential candidate gene located on this region is the retinoid × receptor beta (*RXRB*), which is involved with adipocyte commitment. Epigenetic changes in *RXRB* have been associated with increased IMF deposition without increasing the subcutaneous fat deposition (Wang et al., 2016), which could explain the negative correlation found between *S/P*_*Vx*_ and IMF. This was the only significant region identified for the genetic covariance of *S/P*_*Vx*_ with growth and carcass traits in non-infected purebred sows.

Although not identified in the BiGWAS, the same region on SSC 7 (25.6 to 26.4 Mb) was associated with the genetic covariance between *S/P*_*Vx*_ with TNB in *P_Pure_clean_* and *P_Pure_outbreak_*. Interestingly, the portion of this region outside the MHC has been previously associated with PRRSV susceptibility (Yang et al., 2016) and locates 2 potential candidate genes: the glutamate-cysteine ligase catalytic subunit (*GCLC*) and kelch like family member 31 (*KLHL31*) genes. Interestingly, overlapping windows within this region on SSC 7 (23.6 to 25.9 Mb) contributed with positive and negative genetic covariances in the same analyses for BayesB. This happened for litter mortality traits (i.e., NSB, MUM, and NBD) only. For example, between *S/P*_*Vx*_ and NBD *P_Pure_clean_*, 3 windows on SSC 7 (23.6 to 25.6 Mb) resulted in positive covariance (%Cov = 29%), while 2 windows on SSC 7 (24.8 to 25.9 Mb) resulted in negative covariance (%Cov = −26%) between these 2 traits. Hence, it seems that some SNP in this region have the same direction of effects between S/P and these traits, whereas other SNPs in this region have effects in the oppositive direction. It is important to note that the total genetic covariance between all traits analyzed was also slightly different between BayesA and BayesB. The total genetic covariance in BayesA was overall close to zero, probably due to the strong assumption of this method for having all loci contributing to the genetic covariance between traits, with each locus assuming being sampled from different genetic (co)variances.

These analyses showed that the genomic region on SSC7 (23.6 to 25.9 Mb) is associated with the covariance between *S/P*_*Vx*_ and *S/P_Outbreak_*, as well as between *S/P*_*Vx*_ with litter size traits (i.e., NBA and TNB) in purebred sows (i.e., *P_Pure_clean_* and *P_Pure_outbreak_*). For litter mortality traits, the evidence was not as strong; however, it seems that the direction of SNP effects between S/P ratio and litter mortality traits change within this region, which must have resulted in overall lack of local genetic covariance between these traits. This region is part of the MHC and locates several immune-related genes which are potential candidate genes associated S/P ratio and reproductive performance, simultaneously.

### Effects of major SNP on antibody response and performance traits

Based on our previous study (Sanglard et al., 2020), which identified an effect of the H3GA0020505 SNP on *S/P*_*Vx*_ and reproductive *P*_*Cross_Vx*_ at parities 1 through 3, we assessed the effect of this SNP on *S/P_Outbreak_*, *P*_*Pure_clean*_, and *P*_*Pure_outbreak*_. Also, the effect of the ASGA0032063 SNP, previously associated with *S/P_Outbreak_* using part of these data (Hickmann et al. 2020), was assessed for *S/P*_*Vx*_ and *P*_*Cross_Vx*._

**Figure 5.**
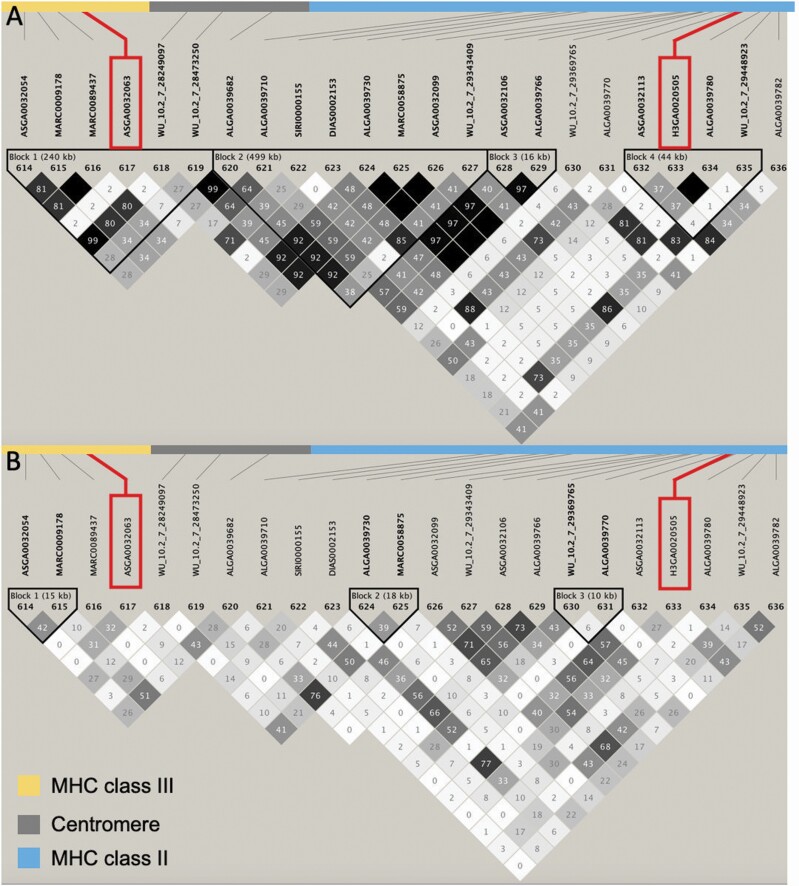
LD analysis of the MHC class III (yellow) and II (blue) on chromosome 7 (24,178,503 to 25,091,206 Mb) for the (A) purebred and (B) crossbred populations. The SNP highlighted in red correspond to the main SNP explaining the genetic variance for antibody response to PRRSV outbreak (ASGA0032063) and vaccination (H3GA0020505). The numbers inside the squares correspond to the *r*^*2*^ (%) measure of LD.

The reason for fitting the 2 SNPs (ASGA0032063 and H3GA0020505) individually and simultaneously is the proximity between the 2 SNPs (0.8 Mb apart), which could result in the 2 SNPs capturing the same QTL. Also, the LD between them was different from zero, showing that they are not independent. The LD for the MHC region, including ASGA0032063 and H3GA0020505 SNP, is demonstrated in Figure 5 for each population. There was moderate LD between these 2 SNPs in the purebred population (*r*^*2*^ = 0.46; Figure 5A), while no LD between them was observed in the crossbred population (*r*^2^ = 0.007; Figure 5B). The moderate LD between these 2 SNPs for the purebred population may explain why the SNP are not significant for *S/P*_Outbreak_ when fitted simultaneously but it was significant for *S/P*_*Vx*_. Other hypothesis that could also explain why the 2 SNPs are not significant when fitted simultaneously would be that there are 2 QTL in LD with each other located between these 2 SNPs, and each SNP is capturing the effect of 1 QTL.

It is interesting to note that ASGA0032063 did not significantly (PPI < 0.70) explain the genetic variance of *S/P*_*Vx*_ in previous analyses using these data (Sanglard et al., 2020). In their study, all SNPs were fitted in the model as random allele substitution effects. However, by fitting SNP as categorical effects, both additive and dominance effects are captured in the model. When we fitted only the allele substitution effect of ASGA0032063 as fixed effect, the allele substitution effect was not significant (*P*-value ≥ 0.07), which is in accordance with the results that this SNP did not significantly (PPI < 0.70) explained part of the genetic variance of *S/P*_*Vx*_.

For performance traits, in general, the heterozygous genotype showed better antibody response and performance for both SNPs. These results agree with what was observed for the effect of the H3GA0020505 SNP on reproductive *P*_*Cross_Vx*_ (Sanglard et al., 2020), in which the AC genotype also showed overall better performance for the traits *S/P*_*Vx*_, NBA, and MUM. Considering that the MHC is a complex region, and selection for this region is controversial (Lavi et al., 2005; Radwan et al., 2020), these results are promising for the possibility of maintaining high genetic variability in this region. Heterozygotes crossbred could be created by fixing the two dam lines for alternate alleles, which would have better performance for S/P ratio to outbreak and vaccination, for body composition in non-infected purebred and in PRRSV-exposed purebred and crossbred sows, and for reproductive traits in non-infected purebred and in PRRSV-exposed purebred and crossbred sows.

## Conclusion

In this study, we showed that *S/P_Outbreak_* and *S/P*_*Vx*_ are highly genetically correlated and have similar genetic control, with genes in the MHC class II region on SSC 7 playing a major role in the genetic covariance between these traits. We also showed that *S/P_Outbreak_* has a favorable genetic correlation with reproductive performance in crossbred sows although low. However, using *S/P_Outbreak_* as a genetic tool is of less interest since *S/P*_*Vx*_ had stronger favorable results: *S/P*_*Vx*_ had favorable genetic correlation with reproductive performance in non-infected purebred sows (by increasing the number of piglets born alive) and in PRRSV-infected purebred sows (by decreasing the number mummified piglets).

Genomic analyses provided novel insights with regards to antibody response and its relationship with reproductive performance. Previous studies have shown associations of haplotypes on the MHC region with reproductive performance (Jung et al., 1989; Vaiman et al., 1998). However, this is the first study to partition the covariance along genomic regions and to identify the proportion of the covariance that is explained by the MHC region. Also, the heterozygote genotype of the H3GA0020505 SNP located within this region was associated with a higher antibody response to PRRSV and better body composition and reproductive performance in non-infected purebred and PRRSV-exposed purebred and crossbred sows. Future work should focus on evaluating the genetic correlation of antibody response to PRRSV vaccination in purebred herds, and to PRRSV outbreak in PRRSV-vaccinated and non PRRS-vaccinated crossbred herds. Also, it is necessary to evaluate the potential costs of implementing antibody response to PRRSV as a selection tool, regarding antibody measurement and genotyping at the commercial level.

## Supplementary Material

skab097_suppl_Supplementary_Figures

skab097_suppl_Supplementary_Table_1

skab097_suppl_Supplementary_Table_2

## Abbreviations

%Cov: proportion of the genetic covariance between 2 traits explained for by a genomic region
ADG: average daily gain
BF: backfat
CG: contemporary group
GBV: genomic breeding values
GRM: genomic relationship matrix
*h*^2^: heritability
IMF: intramuscular fat
ISU: Iowa State University
LD: linkage disequilibrium
LMD: loin muscle depth
MHC: major histocompatibility complex
MUM: number of piglets mummified
MVN: multivariate normal distribution
NBA: number of born alive
NBD: number of born dead
NSB: number of stillborn
*P*_0_: posterior probability
PCA: principal component analysis
P_Cross_Vx_: performance in vaccinated crossbred sows
PCV2: porcine circovirus type 2
PPI: posterior probability of inclusion
*P*_*Pure_clean*_: performance in non-infected purebred sow
*P*_*Pure_outbreak*_: performance in PRRSV-infected purebred sows
PRRS: porcine reproductive and respiratory syndrome
PRRSV: PRRS virus
*r*_g_: genetic correlation
S/P*_Outbreak_*: antibody response to PRRS outbreak
*S/P*_*Vx*_: antibody response to PRRSV vaccination
SLA: swine leukocyte antigen
SSC: *Sus scrofa* chromosome
TGVM: total genetic variance explained by the markers
TNB: total number born
VDL: veterinary diagnostic laboratory
