# Supplementary material for: Genomics of response to porcine reproductive and respiratory syndrome virus in purebred and crossbred sows: antibody response and performance following natural infection vs. vaccination
Source: J Anim Sci. 2021 Mar 29;99(5):skab097. doi: 10.1093/jas/skab097 (PMC8118356; doi:10.1093/jas/skab097)
Supplement: skab097_suppl_Supplementary_Figures [file skab097_suppl_supplementary_figures.docx]

**Supplemental Figures**


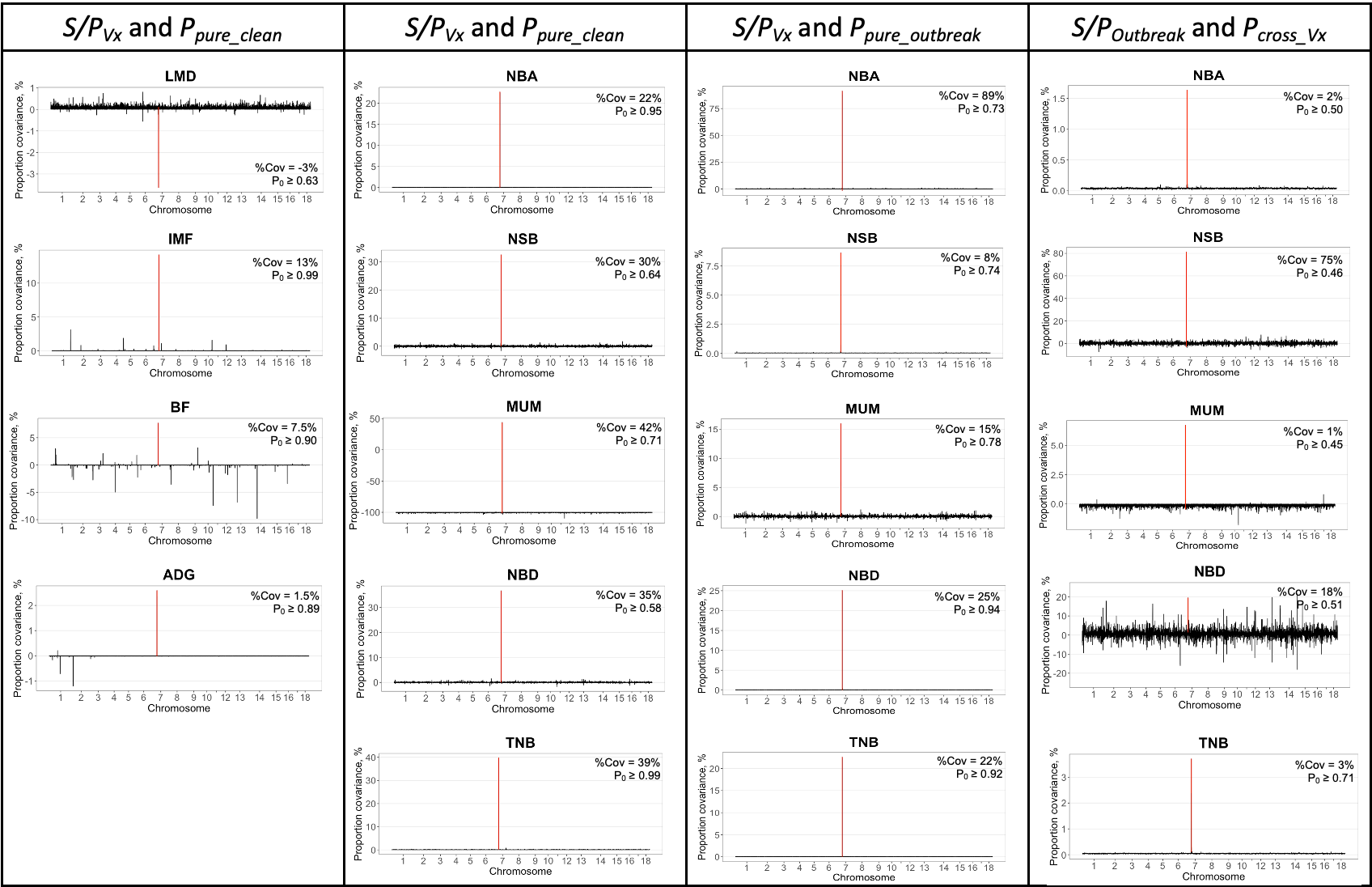


Supplemental Figure 1: Genetic covariance of sample-to-positive (S/P) ratio to Porcine Reproductive and Respiratory Syndrome virus (PRRSV) outbreak (*S/P_Outbreak_*) and PRRSV vaccination (*S/P_Vx_*) with reproductive traits (NBA, number born alive; NSB, number stillborn; MUM, number of mummified; NBD, number born dead; and TNB, total number born) in commercial crossbred sows (*P_Cross_Vx_*), in non-infected purebred sows (*Pure_Clean*), in PRRSV-infected purebred (*Pure_outbreak*), and productive traits (ADG, average daily gain; LMA, loin muscular area; IMF, intramuscular fat, and BF, backfat) using BayesA methodology. The covariance was estimated for 10 SNP moving each 2 SNP. P_0_ corresponds to the lowest posterior probability of the proportion covariance to be greater or smaller than the expected absolute proportion explained by 10 SNP (i.e., 10/28,579 SNP = 0.00035), and %Cov corresponds to the average genetic covariance explained by the SNP windows located on the Major Histocompatibility Complex region (SSC 7; ~23 – 26 Mb), which is highlighted in red.


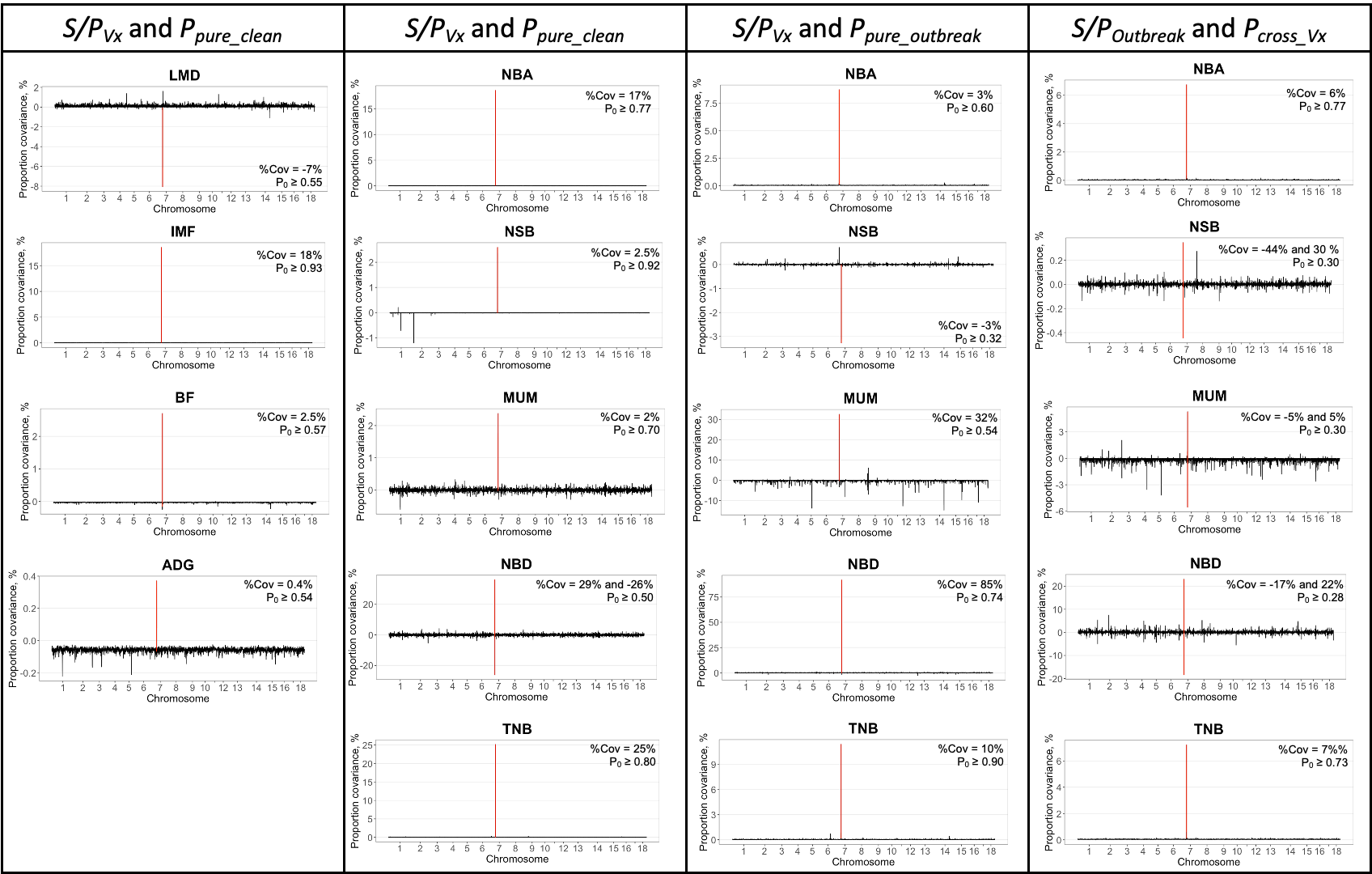


Supplemental Figure 2: Genetic covariance of sample-to-positive (S/P) ratio to Porcine Reproductive and Respiratory Syndrome (PRRS) virus outbreak (*S/P_Outbreak_*) and PRRS vaccination (*S/P_Vx_*) with reproductive traits (NBA, number born alive; NSB, number stillborn; MUM, number of mummified; NBD, number born dead; and TNB, total number born) in commercial crossbred sows (*P_Cross_Vx_*), in non-infected purebred sows (*Pure_Clean*), in PRRS-infected purebred (*Pure_outbreak*), and productive traits (ADG, average daily gain; LMA, loin muscular area; IMF, intramuscular fat, and BF, backfat) using BayesB methodology. The covariance was estimated for 10 SNP moving each 2 SNP. P_0_ corresponds to the lowest posterior probability of the proportion covariance to be greater or smaller than the expected absolute proportion explained by 10 SNP (i.e., 10/28,579 SNP = 0.00035), and %Cov corresponds to the average genetic covariance explained by the SNP windows located on the Major Histocompatibility Complex region (SSC 7; ~23 – 26 Mb), which is highlighted in red.
